# Farnesyl diphosphate synthase regulated endothelial proliferation and autophagy during rat pulmonary arterial hypertension induced by monocrotaline

**DOI:** 10.1186/s10020-022-00511-7

**Published:** 2022-08-12

**Authors:** Tingting Jin, Jiangting Lu, Qingbo Lv, Yingchao Gong, Zhaojin Feng, Hangying Ying, Meihui Wang, Guosheng Fu, Dongmei Jiang

**Affiliations:** 1 Department of Cardiology, Sir Run Run Shaw Hospital, Zhejiang University School of Medicine, 3 East Qingchun Road, Hangzhou, 310016 Zhejiang People’s Republic of China; 2 Key Laboratory of Cardiovascular Intervention and Regenerative Medicine of Zhejiang Province, Hangzhou, China

**Keywords:** Farnesyl diphosphate synthase, Pulmonary artery hypertension, Endothelial, Proliferation, Autophagy, Ras-related C3 botulinum toxin substrate 1

## Abstract

**Background:**

The proliferation ability and autophagy level of pulmonary artery endothelial cells (PAECs) play an important role in promoting the development of pulmonary artery hypertension (PAH), and there is still no effective treatment for PAH. Farnesyl diphosphate synthase (FDPS) is a key enzyme in the mevalonate pathway. The intermediate metabolites of this pathway are closely related to the activity of autophagy-associated small G proteins, including Ras-related C3 botulinum toxin substrate 1 (Rac1). Studies have shown that the mevalonate pathway affects the activation levels of different small G proteins, autophagy signaling pathways, vascular endothelial function, and so on. However, the exact relationship between them is still unclear in PAH.

**Method:**

In vitro, western blotting and mRFP-GFP-LC3 puncta formation assays were used to observe the expression of FDPS and the level of autophagy in PAECs treated with monocrotaline pyrrole (MCTP). In addition, cell proliferation and migration assays were used to assess the effect of FDPS on endothelial function, and Rac1 activity assays were used to evaluate the effect of Rac1 activation on PAEC autophagy via the PI3K/AKT/mTOR signaling pathway. In vivo, the right heart catheterization method, hematoxylin and eosin (H&E) staining and western blotting were used to determine the effect of FDPS on PAEC autophagy and monocrotaline (MCT)-induced PAH.

**Results:**

We show that the expression of FDPS is increased in the PAH module in vitro and in vivo, concomitant with the induction of autophagy and the activation of Rac1. Our data demonstrate that inhibition of FDPS ameliorates endothelial function and decreases MCT-induced autophagy levels. Mechanistically, we found that FDPS promotes autophagy, Rac1 activity and endothelial disfunction through the PI3K/AKT/mTOR signaling pathway.

**Conclusion:**

Our study suggests that FDPS contributes to active small G protein-induced autophagy during MCT-induced PAH, which may serve as a potential therapeutic target against PAH.

**Supplementary Information:**

The online version contains supplementary material available at 10.1186/s10020-022-00511-7.

## Background

Pulmonary artery hypertension (PAH) occurs when the mean pulmonary artery pressure at rest is 25 mmHg or above (Hoeper et al. 2013). Structurally, pulmonary artery vascular remodeling causes lumen occlusion; hemodynamically, pulmonary artery constriction leads to dynamic occlusion of the blood vessels, and all of this contributes to PAH, ultimately resulting in right heart failure (Tuder et al. 2013). With the application of new targeted drugs, the quality of life and time to clinical worsening symptoms and life expectancy of patients have been alleviated to some extent, but the survival rate of patients is still quite low (Thenappan et al. 2018). The mortality rate among pulmonary hypertension patients depends on disease severity, with a 1-year mortality rate of > 10% in high-risk patients, according to an abbreviated version of the risk stratification strategy presented in the 2015 ESC/ERS PH guidelines (Galiè et al. 2015, 2016). The high level of mortality and poor prognosis place a huge burden on patients and health care systems. Many previous studies have enabled us to make progress in recognizing the etiology of PAH. However, it is undeniable that the pathogenesis and clinical treatment of PAH are still obscure and need more research.

Many types of cells take part in the development of PAH, such as vascular cells (endotheliocytes, smooth muscle cells, fibrocytes) and inflammatory cells (Schermuly et al. 2011). Converging studies have shown that endothelial cell (EC) malfunction is one of the primary factors leading to pulmonary artery injury, which plays a great role in maintaining vessel homeostasis (Cool et al. 1999; Spiekerkoetter et al. 2013; Kurakula et al. 2021). Autophagy is a physiological process of cell homeostasis maintenance and self-renewal (Jung et al. 2020). Through autophagy, proteins and organelles within cells can be recycled which is beneficial for cell survival. Previous studies have found increased autophagy levels in idiopathic pulmonary hypertension patients and similar findings have been found in hypoxia-induced pulmonary hypertension mouse models (Lee et al. 2011a, b). Autophagy is associated with primary rat pulmonary artery endothelial cell (PAEC) dysfunction, proliferation and abnormal apoptosis, which are involved in the progression of PAH (Chichger et al. 2019). Studies have confirmed that highly expressed mTOR inhibits the proliferation of PAECs by blocking autophagy and alleviates the progression of hypoxia-induced pulmonary hypertension (Li et al. 2015). However, studies on the autophagy and proliferation of PAECs in monocrotaline induced pulmonary arterial hypertension in rats are not clear, and more research is needed.

Converging studies have indirectly suggested that the mevalonate pathway may contribute to autophagy regulation and endothelial proliferation in pulmonary hypertension (Sakao et al. 2009; Miettinen and Björklund 2015; Zhu et al. 2021). The mevalonate pathway is an important pathway for intracellular cholesterol synthesis, and mevalonic acid is not only a precursor of cholesterol but also a precursor of many nonsteroidal complexes, such as isoprenoids: farnesylpyrophosphate (FPP) and geranylgeranylpyrophosphate (GGPP) (Miziorko 2011). These nonsterol isoprene intermediates play an important role in the posttranslational modification of small G proteins (Ras and Rho families) (McTaggart 2006; Eftekharpour et al. 2017; Seshacharyulu et al. 2019); with the catalysis of isopentenyl transferase, a farnesyl group or geranylgeranyl group was added to the carboxyl terminal of the small G protein and then activated it. Studies have found that some isopentenized small G proteins are very important autophagy related proteins (ATGs) including Rabs, Rheb, RalB, RhoA and Rac1. Therefore, blocking the mevalonate pathway indirectly regulates autophagy through the negative regulation of small G protein activity, but there is no clear answer at home or abroad.

Our previous research indicated that the expression of FDPS was upregulated in monocrotaline (MCT)-induced pulmonary artery hypertension (PAH) rat pulmonary vascular tissue (Jiang et al. 2017). Therefore, we focused on the mevalonate pathway-related enzyme farnesyl pyrophosphate synthase (FDPS) in PAH and investigated whether interference with FDPS can improve damaged endothelial function by affecting the activity of small G proteins and the level of autophagy in the endothelium.

## Methods

### Chemicals and reagents

Type I collagen extracted from rat-tail, Monocrotaline (MCT) and Ibandronate (IB) were purchased from Sigma‐Aldrich (St. Louis, MO, USA). Collagenase II was purchased from Biosharp (Shanghai, China). The Rac1 Activation Assay Biochem Kit™ was acquired from Cytoskeleton (Denver, CO, USA). EGM™-2 BulletKit™ was obtained from Lonza (Walkersville, MD, USA). Anti-FDPS was purchased from Abcam (Cambridge, MA, USA). GAPDH as the loading control was purchased from MultiSciences (Hangzhou, China). Other unspecified antibodies were purchased from Cell Signaling Technology (Beverly, MA, USA).

### Primary rat pulmonary artery endothelial cell (PAEC) isolation, culture and treatment

PAECs were isolated from Sprague–Dawley rats (provided by the experimental animal center of Sir Run Run Shan Hospital, Zhejiang University Medical College) as previously reported (Peng et al. 2013). In brief, we inserted the extracted rat pulmonary artery into the acupuncture needle and flipped it along the needle handle to expose the vascular endothelium to 2 mL of collagenase enzymatic solution (type II, 1800 U/mL). After digestion at 37 °C for 8 min, the cells were resuspended by centrifugation and planted on a small dish that had been coated with collagen type I solution in advance. The cells were then incubated in EBM-2MV with 5% FBS in a humidified atmosphere of 5% CO_2_ and 95% air at 37 °C. IB was dissolved in sterilized phosphate‐buffered saline (PBS, pH 7.4) and stored at − 20 °C. MCT pyrrole (MCTP) was prepared by the method described previously (Mattocks et al. 1989).

### PAH rat model

This study followed the National Institutes of Health Guidelines for the Care and Use of Laboratory Animals and was approved by the Animal Welfare Ethics Committee of Sir Run Run Shaw Hospital affiliated Zhejiang University (SRRSH202002233). Male Sprague–Dawley male rats (8-weeks old; weighing 200 ± 10 g) were provided by the experimental animal center of Sir Run Run Shaw Hospital, Zhejiang University Medical College. Three rats were kept in each cage at 22 °C, with light and darkness cycling for 12 h and free access to drinking water and a normal diet. After a single intraperitoneal injection of MCT (60 mg/kg), the PAH rat model was induced after 4 weeks of a routine diet (West and Hemnes 2011). For the IB treatment group, a single subcutaneous injection of IB (28 μg/kg) was given 2 weeks after MCT injection (De La Piedra et al. 2011).

Rats were randomly divided into the PAH group (n = 4), IB treatment group (n = 10) or control group (n = 10). The rat belonging to the control group were injected with a corresponding dose of normal saline once, and all rats were sacrificed 28 days later.

### Histological analysis

The lung tissue specimens were fixed in 4% paraformaldehyde for 24 h and cut into 4 μm-thick paraffin sections. After staining with hematoxylin and eosin, we used a microscope (Olympus CX23 Binocular Microscope, Tokyo, Japan) to observe the stained sections. Pulmonary remodeling was assessed by calculating the thickness of the media of the distal pulmonary vessels (< 100 μm in diameter). The ratio of the medial wall area (the area between the inner and outer plates) to the vascular area (the area between the outer plates) represents the degree of pulmonary remodeling.

### Hemodynamic parameters

After weighing, the rats were injected with 3% pentobarbital sodium (0.1–0.2 mL/100 g) and fixed on the anatomical table. A PE50 catheter was inserted into the right external jugular vein into the right ventricle and the right ventricular systolic pressure (RVSP) was measured and recorded by a MedLab Biological signal acquisition and processing system (Nanjing Medease Science and Technology, Nanjing, China).

### Right ventricular hypertrophy (RVH) measurement

After measuring RVSP, the rat was sacrificed, and the heart was isolated and washed with precooled PBS. The ventricles were divided into two parts: one is right ventricle (RV), the other is left ventricle (LV) and interventricular septum (IVS). Right ventricular hypertrophy was evaluated by the ratio of RV weight to (LV + IVS) weight.

### Cell proliferation assay

To identify the viability and proliferation of PAECs, we used a Cell Counting Kit-8 (CCK-8; Dojindo, Kumamoto, Japan). In short, PAECs were grown in 96-well plates at a density of 4 × 10^4^ cells per well. After MCTP treatment at the specified dose and time, 10 μL tetrazolium salt was dropped into each well and cultured in an incubator for another 2 h. Absorbance at 450 nm was measured using a spectrophotometer (Bio Tek, VT, USA), and cell viability was calculated according to the kit instructions.

### Cell migration assay

Transwell migration assays were performed using Millicell® Hanging Cell Culture Insert (Millipore, St. Louis, MO, USA) with a filter with a pore size of 8 μm. The 8 × 10^4^ cells were suspended in the corresponding treatment group of medium and planted into the upper chamber of the insert and EBM-2MV with 5% FBS was added to the lower chamber. After 24 h of culture in the incubator, the cells were fixed with methanol and stained with crystal violet. The cells on the upper surface of the membrane were gently erased with cotton swabs, and the cells crossing to the lower surface of the membrane were observed under Olympus DP72 microscope (Olympus, Tokyo, Japan). Four random fields were taken from each well, and the number of migrating cells was counted to measure the migrating ability of cells.

### Wound healing assay

The PAECs were plated in 6-well plates with an adherent density of about 70%. After overnight culture, the PAECs were covered the plate. Then created a wound by manually scraping the cell monolayer with a 200 μL pipetting tip. in each well, washed with PBS three times to remove cell debris and floating cells, and incubated at 37 °C and 5% CO_2_ with medium containing the corresponding treatment reagent. Images were taken at the same position under a microscope before and after the 24 h incubation. ImageJ software was used to measure cell migration ability. The average scratch width is the ratio of the scratch gap area to the scratch length. The migration index = (0 h scratch width − 24 h scratch width)/0 h scratch width × 100%.

### Immunoblotting analysis

Cells and tissues were scraped and lysed in radioimmunoprecipitation assay lysis buffer with 1 mM phenylmethylsulfonylfluoride. Proteins were quantified by a BCA Protein Assay Kit (Beyotime, Shanghai, China), separated using SDS-PAGE and transferred to PVDF membranes (Bio-Rad, Hercules, CA, USA). After blocking with 5% nonfat dry milk for 1 h, the bands were recognized by the indicated primary antibodies overnight followed by horseradish peroxidase-conjugated secondary antibody for another 2 h. Protein bands covered by enhanced chemiluminescence were visualized on the BioRad ChemiDoc MP Imaging System.

### Quantitative real-time polymerase chain reaction (qRT-PCR)

Pulmonary artery RNA was extracted by the Tissue RNA Purification Kit Plus (ES Science).and reverse transcribed into complementary DNA with a PrimeScript RT reagent Kit (Perfect Real Time) (Takara, Tokyo, Japan). qRT-PCR was performed with Hieff® qPCR SYBR® Green Master Mix (No Rox) (Yeasen, Shanghai, China) on a LightCycler 480 (Roche). The fold change in relative mRNA expression was calculated using the 2^−ΔΔCt^ method, with 18S RNA as an internal control. The primers for rat FDPS were forward: GCACGCTCTGCTTTTAGGTG, reverse: GTGCTTCAACGCGGATTCTC. The primers for rat Beclin1 were forward: GAATGGAGGGGTCTAAGGCG, reverse: CTTCCTCCTGGCTCTCTCCT. The primers for rat Rac1 were forward: GAGACGGAGCCGTTGGTAAA, reverse: TGGGGATGTACTCTCCAGGG. The primers for rat 18S were forward: AAACGGCTACCACATCCAAG, reverse: CCTCCAATGGATCCTCGTTA.

### Rac1 activity assay

PAECs were untreated or treated with either MCTP (50 μm, 24 h) or IB (100 μm, 24 h) prior to harvesting. The level of GTP-bound Rac1 was measured using a Rac1 Activation Assay Biochem Kit™ according to the manufacturer's instructions.

### mRFP-GFP-LC3 puncta formation assays

A tandem mRFP-GFP-LC3 adenovirus system (HanBio, Shanghai, China) was used to detect autophagy under a Leica confocal microscope (Leica Microsystems, Wetzlar, Germany). PAECs were planted in a 35 mm confocal dish at a density of 10^6^ cells per dish, and Ad-mGFP-GFP-LC3 was transfected into PAECs. After the indicated treatment, fluorescence-positive cells were detected under a fluorescence confocal microscope. The red spots are autolysosomes (mRFP), and the yellow spots are autophagosomes (RFP + GFP). Since GFP fluorescent protein is sensitive to acidity, GFP fluorescence will quench after fusion of autophagosomes and lysosomes, and only red fluorescence can be detected at this time. Therefore, the level of autophagic flow can be clearly seen by counting spots of different colors.

### Transfection of small interfering RNA (siRNA)

For knockdown of target genes, we obtained siRNAs from Sigma-Aldrich (St. Louis, MO). The target sequence for the FDPS sense strand was 5′-CUCAGCAGCGCCAGAUCUUdTdT-3′ and was 5′-GCUAUCUACCGCCUGCUUAdTdT-3′. The target sequence for the Rac1 sense strand was 5′-CCAAUACUCCCAUCAUCCUdTdT-3′ and was 5′-AGGAUGAUGGGAGUAUUGGdTdT-3′. The PAECs were transfected with siRNA using Lipofectamine-RNAiMax (Invitrogen, Carlsbad, CA, USA).

### Detection of NO concentration

The concentration of NO released from the PAECs in the culture supernatant and cell lysates was measured after 24 h of MCTP and/or IB treatment using a commercial NO assay kit (Beyotime Biotechnology, Shanghai, China) according to the manual.

### Statistical analysis

All data are expressed as the mean ± SEM. Experiments were repeated at least three times. Comparisons between two groups were performed using Student’s t-test, and one-way ANOVA was used for comparisons between multiple groups. There was a statistically significant difference when P < 0.05.

## Results

### PAH increased the expression of farnesyl diphosphate synthase (FDPS) in vivo and in vitro

**Fig. 1:**
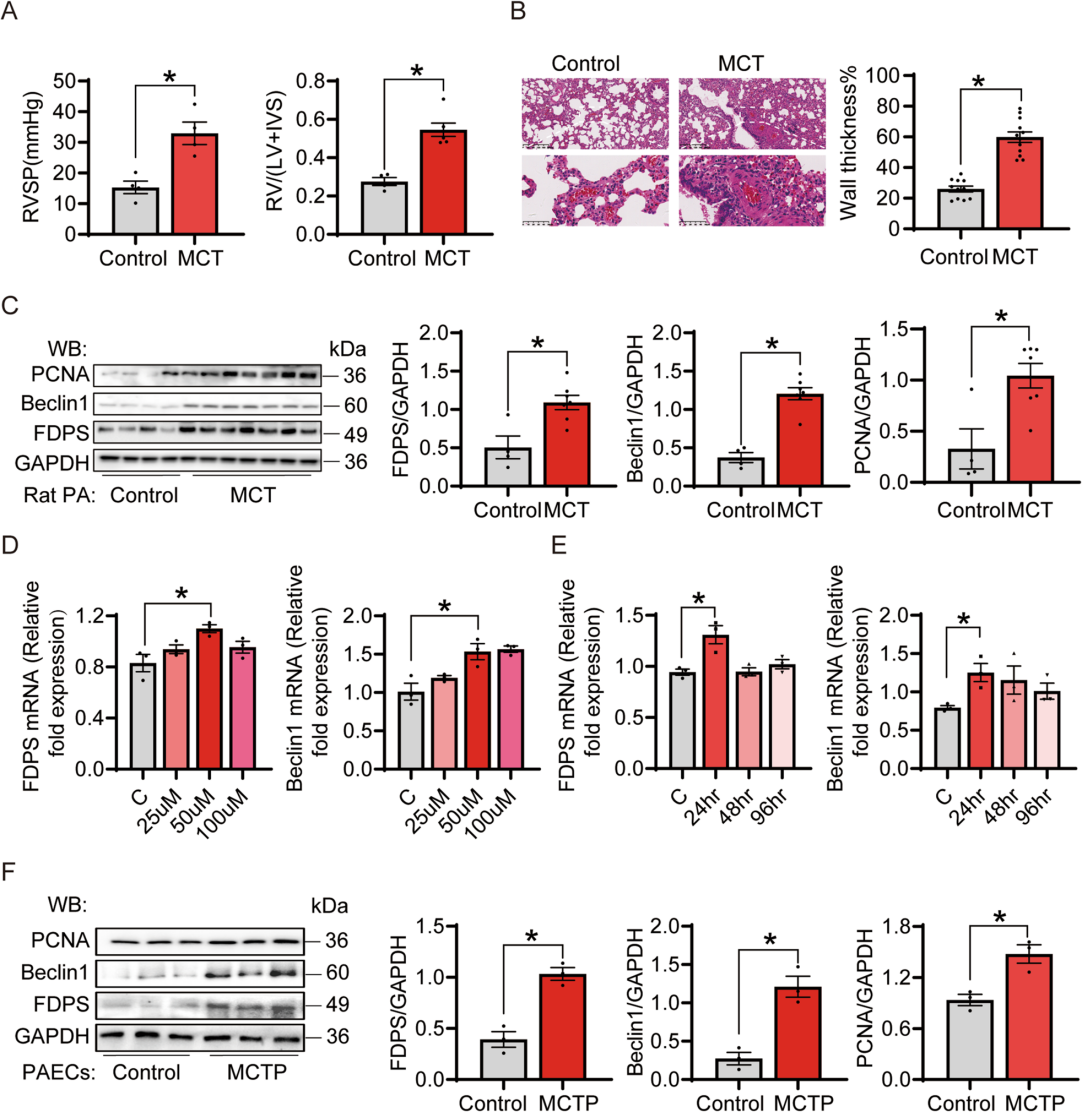
PAH increased the expression of FDPS, PCNA and Beclin1 in vivo and in vitro. **A** RVSP and RV/(LV + IVS) in control and MCT groups. n = 4–6 per group. **B** Hematoxylin and eosin staining of lung tissues. The index of wall thickness was calculated and showed in right. Scale bar = 50 μm. n = 11–12 per group. **C** Immunoblot analysis for FDPS, PCNA and Beclin1 in control and MCT-induced PAH rats. GAPDH was used as internal reference. n = 4–7 per group. **D** The mRNA level of FDPS and Beclin1 in PAECs after different concentration of MCTP treatment for 24 h was detected. n = 3 per group. **E** The mRNA level of FDPS and Beclin1 in PAECs after 50 μm MCTP treatment for different times was detected. Quantitative RT PCR (qRT-PCR) was conducted to measure mRNA level changes, with 18S as an internal control. n = 3 per group. **F** Immunoblot analysis for FDPS, PCNA and Beclin1 in the control and MCTP treatment PAECs. GAPDH was used as internal reference. n = 3 per group. Data are expressed as the mean ± SEM. All experiments were independently replicated in triplicate. *P < 0.05

FDPS, a key enzyme in the mevalonate (MEV) pathway, plays an important role in vascular stiffness and angiogenesis (Han et al. 2016; Chichger et al. 2019), which suggests that it may affect pulmonary hypertension. We established a rat pulmonary hypertension model by injection of monocrotaline (MCT 60 mg/kg) once (Colvin and Yeager 2014). The RVSP of the PAH group as well as the ratio of RV weight to (LV + IVS) weight were higher than those in the control (Fig. 1A). Pulmonary vascular remodeling is a significant pathological feature of PAH. The micropulmonary arteries (less than 100 μm in diameter) were significantly thickened in the pulmonary hypertension group (Fig. 1B). In addition, we observed significant fibrosis in the right heart tissue of the PAH rats by Masson and Sirius red staining (Additional file 1: Fig. S1A). These results indicated that we successfully constructed a pathological model of pulmonary hypertension in rats. We found that, in agreement with our previous study (Jiang et al. 2017), PAH rat pulmonary artery tissue showed a significant increase in FDPS expression at the protein level, accompanied by upregulation of proliferating cell nuclear antigen [PCNA, a good cell proliferation marker (Almendral et al. 1987)] and Beclin1 protein (Fig. 1C).

For the in vitro PAH module, we isolated primary rat pulmonary artery endothelial cells (PAECs) from Sprague–Dawley rats. PAECs were identified by immunofluorescence by detecting the presence of von Willebrand factor (Additional file 1: Fig. S1B). Then, we treated the PAECs with MCT pyrrole (MCTP) to simulate an in vitro model of PAH. MCTP is the hepatic metabolite of MCT in vivo, which then can result in a series of pulmonary pathological changes. Therefore, in the in vitro model, pulmonary artery endothelial cells were directly treated with MCTP to simulate PAEC injury in MCT-induced pulmonary hypertension rats. The migration function was enhanced in MCTP-treated PAECs (Additional file 1: Fig. S1C), and NO production was decreased by MCTP treatment (Additional file 1: Fig. S1D), which was consistent with the phenotypic changes in endothelial cells in the in vivo model of PAH. We detected the mRNA expression of FDPS under different MCTP concentrations and treatment time conditions and found that the level of FDPS significantly increased when treated with 50 μM MCTP for 24 h, as did the expression of Beclin1 (Fig. 1D, E). In subsequent experiments, cells were treated with this condition. In contrast, the mRNA levels of other types of enzymes in the MEV pathway, including farnesyltransferase β (FNTB) and HMG-CoA reductase (HMGCR), showed no significant changes (Additional file 1: Fig. S1E). Farnesyltransferase α (FNTA) was also elevated in the MCTP group; however, we preferred to study the upstream, key enzyme FDPS. PAECs subjected to 50 μM MCTP for 24 h showed a significant increase in FDPS, Beclin1 and PCNA expression at the protein level (Fig. 1F). In summary, these findings suggest that PAH leads to upregulation of FDPS and induction of autophagy and proliferation.

### Inhibition of FDPS improved pulmonary artery remodeling in PAH rats

**Fig. 2:**
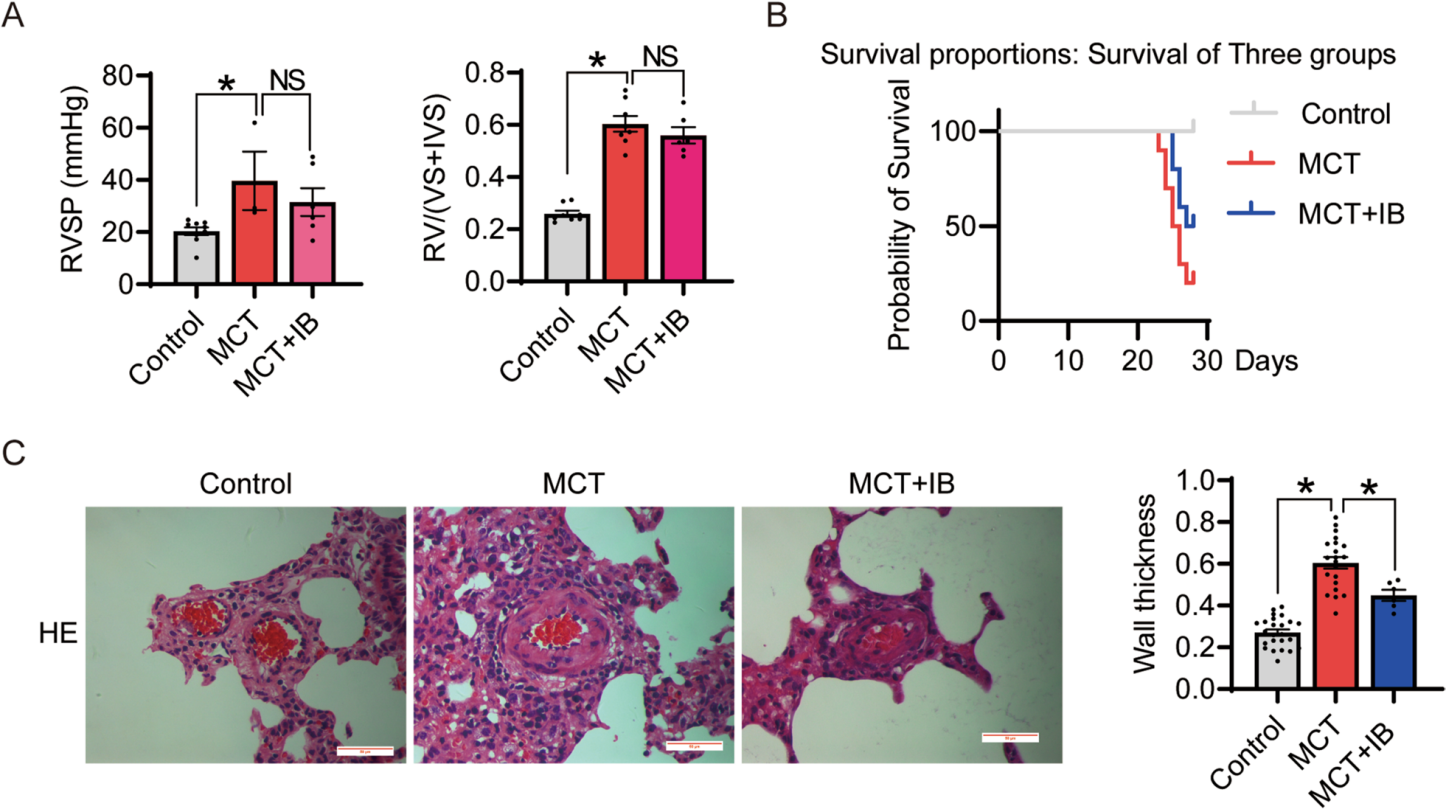
Effect of ibandronate (IB) treatment on MCT induced rat PAH module. **A** RVSP and RV/(LV + IVS) in control, PAH and IB treated groups. n = 3–9 per group. **B** The probability of survival in the three groups. n = 4–10 per group. **C** Hematoxylin and eosin staining of lung tissues. The index of wall thickness was calculated and showed in right. n = 6–24 per group. Scale bar = 50 μm. Data are expressed as the mean ± SEM. All experiments were independently replicated in triplicate. *P < 0.05

To investigate the function of FDPS in vivo, MCT induced PAH rats were subjected to ibandronate (IB), a chemical inhibitor of FDPS. IB is a third-generation bisphosphonate that acts as an analogue of isoprene diphosphate lipids, thereby inhibiting FDPS (Dooley and Balfour 1999). High doses of IB are commonly used for the treatment of bone-related diseases. Research has shown that low-dose ibandronate significantly reduces endothelial cell growth (Morgan et al. 2009). We used ibandronate to inhibit the FDPS enzyme in vivo and in vitro to investigate the role of FDPS in pulmonary artery endothelial cell injury in pulmonary hypertension. Although treatment with ibandronate did not improve hemodynamic changes in the rats with PAH (Fig. 2A), we found an upward trend in survival, we thought that there was no significant difference because of the small sample size (Fig. 2B), and an effective reduction in pulmonary fine arterial remodeling (Fig. 2C). Taken together, our data indicate that inhibition of FDPS protects rats against pulmonary artery remodeling in MCT induced PAH.

### Inhibition of FDPS improved PAECs phenotype induced by MCTP in vitro

**Fig. 3:**
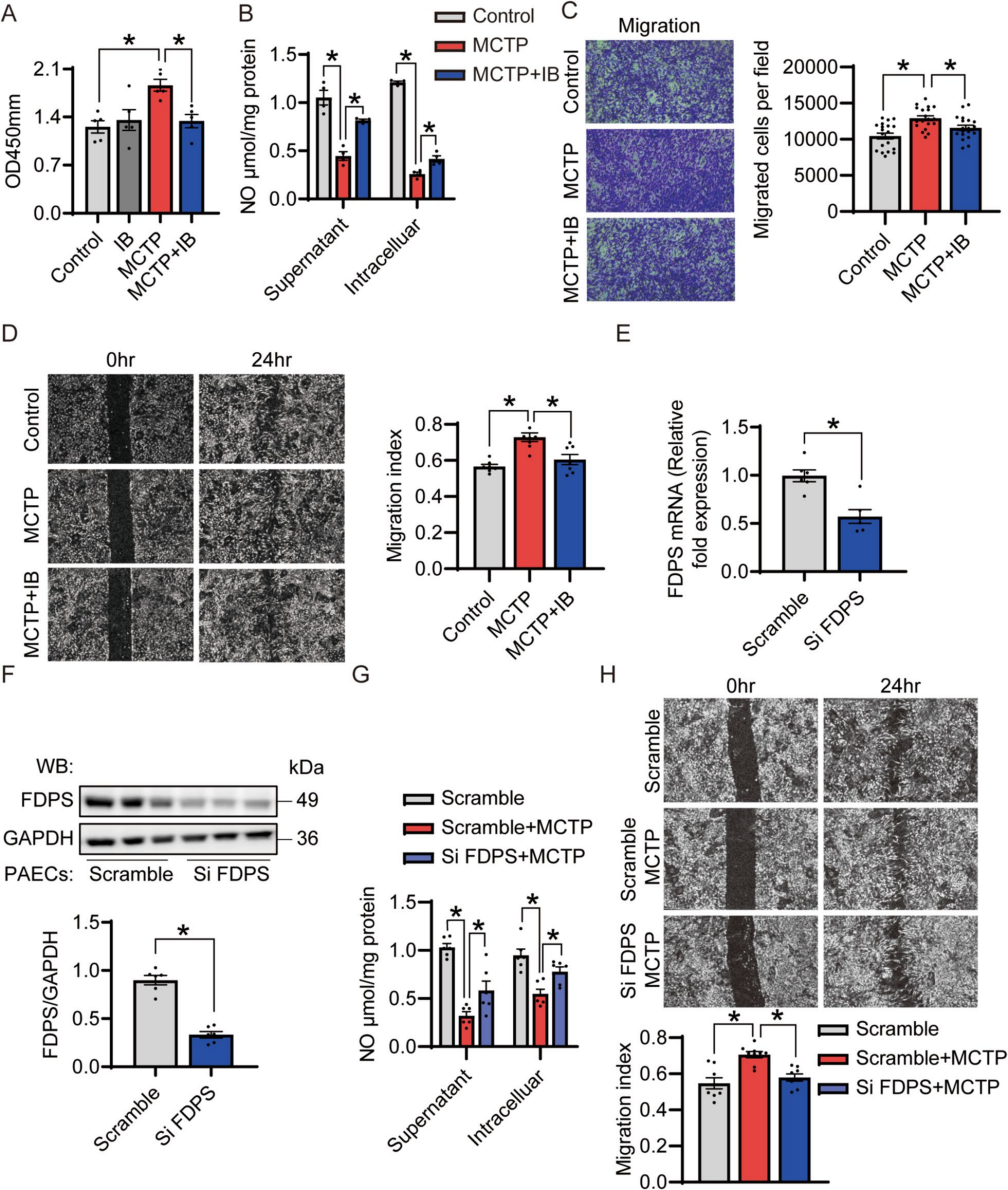
Inhibition of FDPS decreased the proliferation and migration of MCTP-induced PAECs, increased the NO level in MCTP-induced PAECs. **A** CCK8 assay on cell proliferation at PAECs. n = 5 per group. **B** NO assay was performed to detect NO concentrations in culture medium and cell lysates. Relative NO concentration in cell lysates was normalized based on total cellular protein concentrations. n = 4 per group. **C** Crystal violet staining of PAECs that crossed the polycarbonate membrane of the Transwell invasion chamber. Representative photomicrographs were shown in left. The number of migrated cells were calculated and showed in right. n = 19–20 per group. **D** The scratch assay following 50 μM MCTP and 100 μM IB treating for 24 h in PAECs. Representative photomicrographs were shown in left. The index of migration was calculated and showed in right. n = 7–8 per group. **E** The relative gene expression of FDPS decreased in the siFDPS‐transfected PAECs. qRT-PCR was conducted to examine mRNA level changes with 18S as an internal control. n = 6 per group. **F** Immunoblot analysis for FDPS. GAPDH was used as internal reference. n = 6 per group. **G** Knockdown of FDPS elevated NO production of MCTP-induced cell. n = 6 per group. **H** The scratch assay following 50uM MCTP treating for 24 h in PAECs and si FDPS-transfected PAECs. Upper showed in vitro scratch assay. The index of migration was calculated and showed in below. n = 8–9 per group. Data are expressed as the mean ± SEM. All experiments were independently replicated in triplicate. *p < 0.05

To explore the role of FDPS in PAH in vitro, we adopted an inhibitor-based approach. First, we treated PAECs with 100 μM IB, a potent and irreversible FDPS inhibitor (Gadelha et al. 2020), along with MCTP for 24 h. Our results showed that inhibition of FDPS significantly reduced cell proliferation caused by MCTP (Fig. 3A). The NO assay verified that IB treatment elevated MCTP induced supernatant and intracellular NO production (Fig. 3B), which was mainly because of the reduced endothelial NO synthase (eNOS) activity. Additionally, the decreased number of migrated cells in the IB-treated group confirmed that the inhibition of FDPS relieved PAECs dysfunction due to PAH (Fig. 3C, E). To further test the function of FDPS in PAH directly, we transfected PAECs with si FDPS to knock down FDPS. Si FDPS efficiently decreased target gene expression at both the protein and mRNA levels (Fig. 3E, F). Knockdown of FDPS modestly but significantly increased NO production in the supernatant and intracellular (Fig. 3G) and the migration function of PAECs was in remission (Fig. 3H). Collectively, these results demonstrate that inhibition of FDPS protects PAECs from MCTP treatment in vitro.

### The effect of FDPS inhibitors is mainly achieved by reducing autophagy

**Fig. 4:**
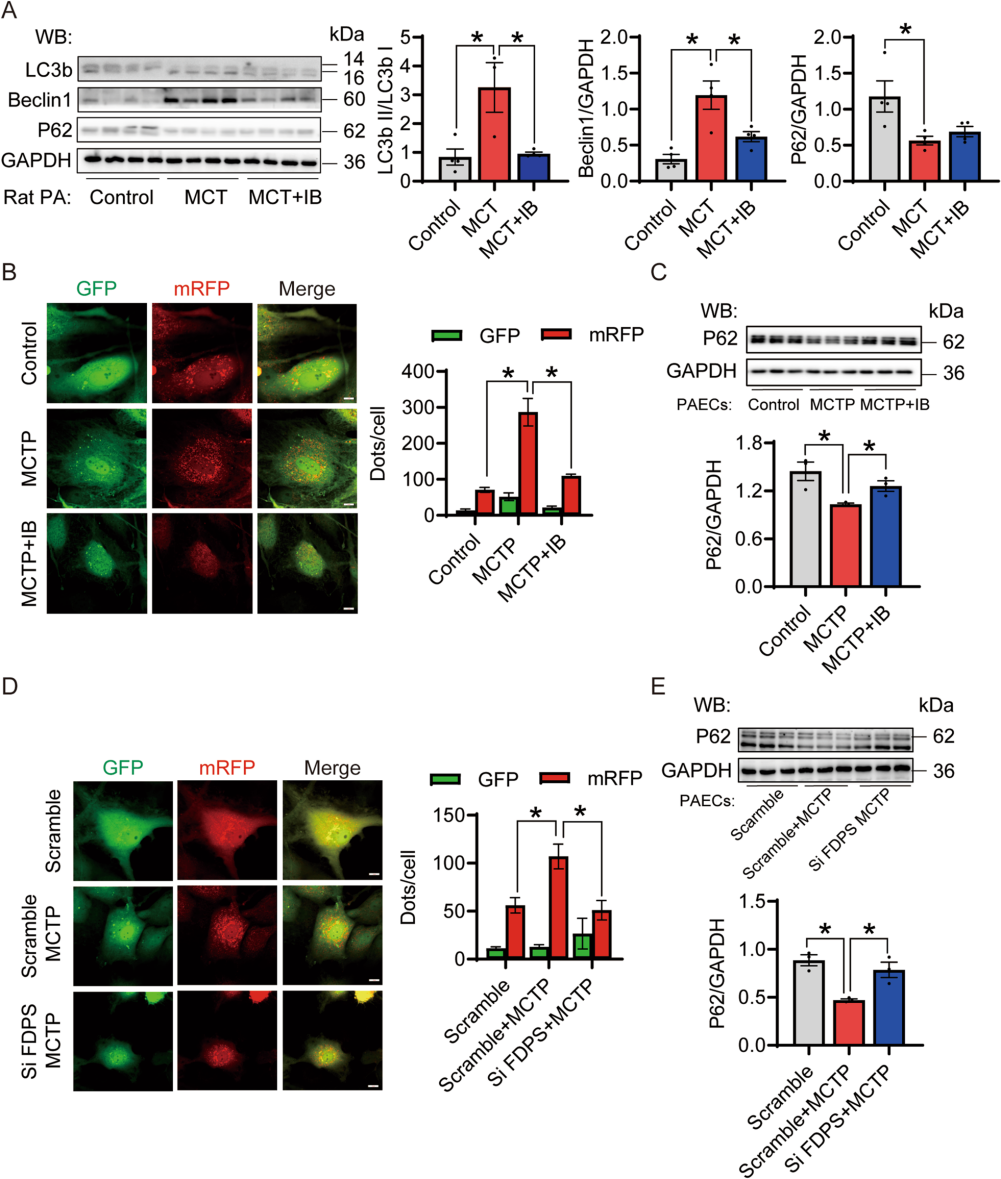
Inhibition of FDPS decreased the autophagy level in vivo and in vitro PAH module. **A** Immunoblot analysis for LC3b, Beclin1 and P62 in the control, MCT-induced PAH rats and IB treated PAH rats. GAPDH was used as internal reference. n = 4 per group. **B** Inhibition of FDPS decreased the MCTP induced autophagy in PAECs. PAECs transfected with Ad-mRFP-GFP-LC3 was observed and analyzed by microscopy. The representative images are displayed at left. Scale bars = 20 μm. The numbers of green (GFP signal) and red (mRFP signal) puncta per cell were counted. n = 7–8 per group. **C** Immunoblot analysis for P62 in the control, MCTP treated PAECs and IB treated PAECs. GAPDH was used as internal reference. n = 3 per group. **D** Knockdown FDPS decreased the MCTP induced autophagy in PAECs. The representative images are displayed at left. Scale bars = 20 μm. The numbers of green (GFP signal) and red (mRFP signal) puncta per cell were counted. n = 7–9 per group. **E** Immunoblot analysis for P62 in the scramble, MCTP treated scramble PAECs and IB treated siFDPS transfected PAECs. GAPDH was used as internal reference. n = 3 per group. Data are expressed as the mean ± SEM. All experiments were independently replicated in triplicate. *p < 0.05

Previous work suggests that autophagy is involved in PAH (Teng et al. 2012). Since isoprenoids produced by the mevalonate pathway activate small G proteins, some of which are autophagy-related proteins (ATGs) including Rac1 leading to autophagy, we hypothesized that the inhibition of FDPS might alleviate PAH damage by reducing autophagy. Indeed, the expression of autophagy-associated proteins, such as LC3b and Beclin1, in MCT-induced PAH rat pulmonary artery tissue after IB treatment was downregulated, and the expression of P62 was increased (Fig. 4A). In vitro, we observed that the red dots (mCherry signal) were markedly reduced in the IB-treated and si FDPS-transfected PAECs under MCTP treatment (Fig. 4B, D). Moreover, inhibition of FDPS increased the expression of P62 in MCTP-treated PAECs (Fig. 4C, E).

### FDPS contributed to small G protein-induced autophagy during PAH through the PI3K/AKT/mTOR signaling pathway

**Fig. 5:**
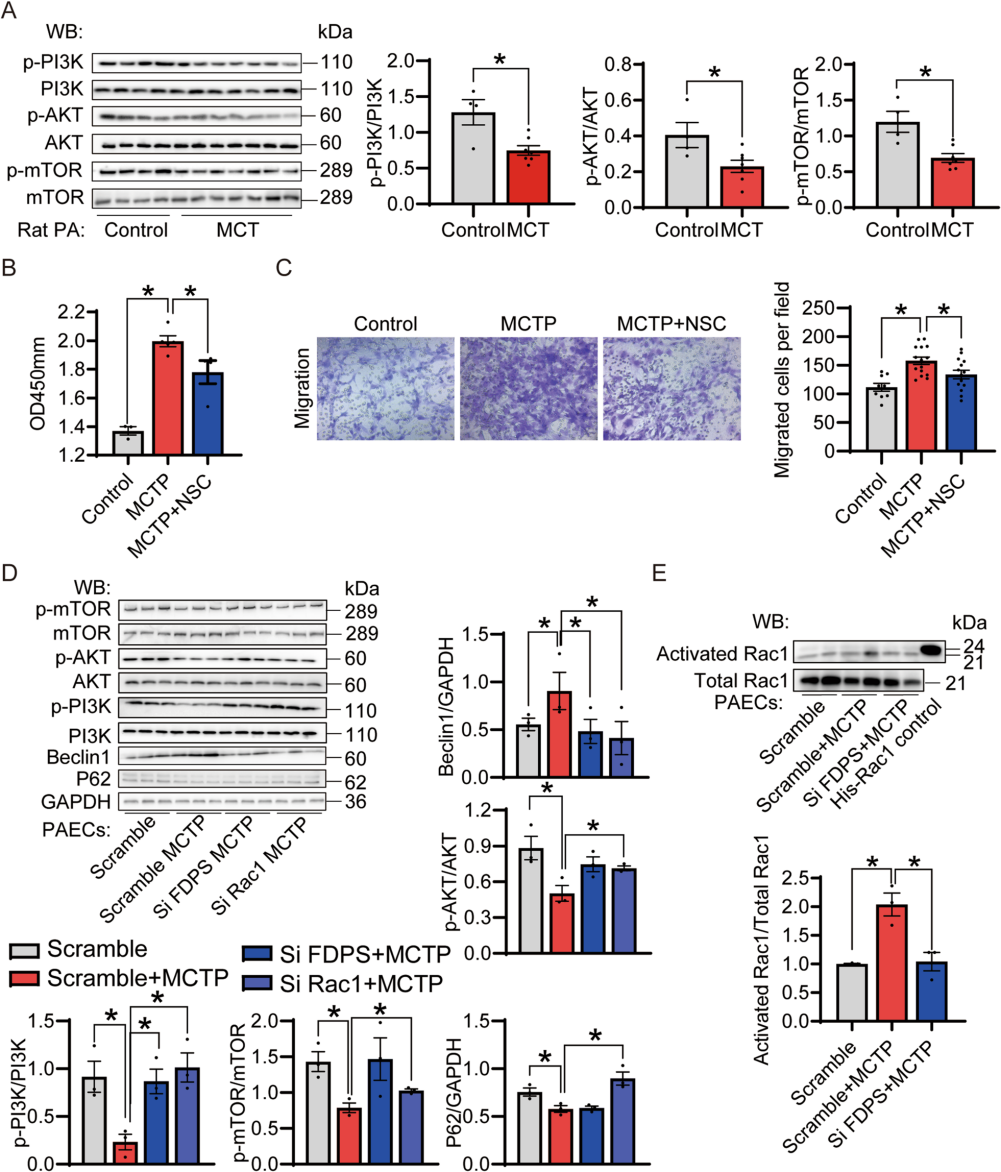
The effect of FDPS and Rac1 on PAH through PI3K/AKT/mTOR pathway. **A** Immunoblot analysis for p-AKT, AKT, p-mTOR, mTOR, P-PI3K and PI3K in the normal, MCT-induced PAH rats. n = 4–9 per group. **B** Pretreated with selective Rac1 inhibitor NSC23766 (NSC) 100 μm for 3 h significantly reduced MCTP induced PAECs proliferation. CCK8 assay on cell proliferation at PAECs. n = 4–5 per group. **C** Pretreated with selective Rac1 inhibitor NSC23766 (NSC) 100 μm for 3 h significantly reduced MCTP induced PAECs migration. Crystal violet staining of PAECs that crossed the polycarbonate membrane of the transwell invasion chamber. Representative photomicrographs were shown in left. The number of migrated cells were calculated and showed in right. n = 9–14 per group. **D** Knockdown of Rac1 and FDPS reduced the autophagy level in the MCTP treated PAECs through PI3K/AKT/mTOR pathway. Immunoblot analysis for p-AKT, AKT, p-mTOR, mTOR, p-PI3K, PI3K, LC3b II and P62 in the scramble, MCTP treated scramble, MCTP treated siFDPS-transfected and MCTP treated siRac1-transfected PAECs. GAPDH was used as internal reference. n = 3 per group. **E** Western bolt was used to detect the total and activated Rac1 using a pull-down assay. n = 3 per group. Data are expressed as the mean ± SEM. All experiments were independently replicated in triplicate. *p < 0.05

Some studies have found that inhibiting FDPS can reduce the activation of Rac1 (Han et al. 2016; Dai et al. 2017), which is closely associated with the PI3K/AKT/mTOR pathway (Saci et al. 2011). The PI3K/AKT/mTOR pathway, an important intracellular signaling cascade, comprising three core molecules, PI3Ks, AKT and mammalian target of rapamycin (mTOR), is directly related to cell growth, proliferation and metabolic regulation (Yu and Cui 2016). To determine the mechanism of action of FDPS in PAH, immunoblot analysis was conducted. MCT increased the expression of p-PI3K, p-AKT and p-mTOR in the PAH rat group in vivo (Fig. 5A). CCK8 and Transwell assays were next used to investigate whether pretreatment with 100 μM NSC23766 (NSC), a Rac1 inhibitor, for 3 h reduced MCTP-induced PAECs proliferation and migration (Fig. 5B, C). We found that the proliferation and migration of PAECs caused by MCTP were mitigated under the NSCs. To further test the function of Rac1 in PAH directly, we transfected PAECs with siRac1 to knock down Rac1. SiRac1 efficiently decreased target gene expression at the protein level (Additional file 1: Fig. S2a). Knockdown of Rac1 significantly reduced the PI3K/AKT/mTOR signaling pathway and autophagy-associated proteins (Fig. 5D), and a similar result was observed after knocking down FDPS. Moreover, through a pull-down assay, we found that knockdown of FDPS reduced the Rac1 activity induced by MCTP treatment. In our WB experiment results, knocking down FDPS and Rac1 can both reduce the increase in MCTP-induced autophagy, but knocking down FDPS does not lead to stable and significant changes in the PI3K/AKT/mTOR pathway, similar to knocking down Rac1. We believe that Rac1 is downstream of FDPS, which regulates the classical autophagy signaling pathway by regulating the activity of Rac1. However, due to the correlation and complexity of biological signals, although Rac1 activity is significantly downregulated after FDPS knockout, other signaling pathways may change during this process, making the result not as perfect as that of direct Rac1 knockout. Furthermore, knocking down FDPS may not downregulate Rac1 activity as much as directly knocking down Rac1 to reduce the activation of Rac1. Of course, more experiments are needed to further explore the specific mechanism. Collectively, these findings suggest that PAH induced FDPS expression and enhanced Rac1 activity, which led to high autophagy levels through the PI3K/AKT/mTOR signaling pathway.

## Discussion

PAH causes significant morbidity and mortality and significantly reduces quality of life and death in ~ 25% to 60% of patients 5 years after diagnosis (Barst et al. 2012; Galiè et al. 2016). In our study, we assessed the effects of FDPS interfering with MCT-induced pulmonary artery remodeling in vivo and investigated the possible mechanism. Thus, we demonstrate that inhibition of FDPS alleviates MCT-induced pulmonary remodeling by reducing Rac1 activation, in which the PI3K/AKT/mTOR signaling pathway also participates.

We constructed a commonly used rat model of PAH by intraperitoneal or subcutaneous injection of MCT (usually 60 mg/kg) once and PAH developed 3–4 weeks later (Campian et al. 2006). In 1967, Kay et al. found that feeding *C. spectabilis* seeds to rats could induce PAH, and monocrotaline (MCT) was their active chemical component (Kay et al. 1967). Over the past 50 years, the MCT-induced rat pulmonary artery hypertension model has been widely used by researchers to study the animal pathological model of the occurrence and development of PAH due to its repeatability, ease of operation and low cost (Gomez-Arroyo et al. 2012). The choice of the animal model is mainly dictated by the scientific question to be answered. MCT has been reported to damage pulmonary arterial endothelial cells (PAECs) and cause PAH (Rosenberg and Rabinovitch 1988), but the exact toxicological mechanism remains unclear. In our study, pulmonary artery endothelial cells were the main research object, so we chose this animal model to study the potential mechanism of PAH. The MCT-induced PAH rat model with high mortality is comparable to human PAH in terms of hemodynamic and histopathological severity, moreover, this model is conducive to researching the pathomechanism of PAH (Naeije and Dewachter 2007). Although the pathologic factors of MCT-induced PAH differ from those of human PAH (Nogueira-Ferreira et al. 2015), considering that in this study, we focused on vascular remodeling, endothelial cell injury, and the repeatability, ease of operation and low cost of this model, we believe that the MCT model is suitable for our study, which can be used to elucidate the role of pulmonary artery endothelial cells in PAH development and its related molecular and cellular pathways, and hopefully find new therapeutic methods.

The mevalonate pathway has a great effect on cell proliferation, metabolism and survival (Goldstein and Brown 1990; Saeedi Saravi et al. 2017), especially in tumor cells (Likus et al. 2016; Mullen et al. 2016). We believe that the phenotypic characteristics of endothelial cells in pulmonary hypertension are somewhat similar to those of cancer cells, so we focused on the effect of the MVA pathway during PAH. Metabolites of the MVA pathway affect lung resident cell homeostasis not only (Murphy et al. 2008) by regulating the integrity of the barrier formed by endotheliocytes (Jacobson et al. 2005; Chen et al. 2008b), proliferation of smooth muscle cells (Vigano et al. 1995; Takeda et al. 2006), accumulation of extracellular matrix (Schaafsma et al. 2011), and cytokine production in epithelial cells (Sakoda et al. 2006; Zeki et al. 2012), but also by participating in important physiopathological reactions, including endoplasmic reticulum stress (Chen et al. 2008a), autophagy (Zhang et al. 2013) and apoptosis (Ghavami et al. 2011) and eventually result in pathophysiological changes in the pulmonary artery. FDPS catalyzes the generation of geranyl pyrophosphate (GPP) and farnesyl pyrophosphate (FPP) in the mevalonate pathway. In addition to participating in the biosynthesis of cholesterol, FPP promotes the activation of small GTPases. Inhibition of FDPS ameliorates chronic cardiac remodeling caused by excessive pressure load (Zhao et al. 2016). We observed upregulation of FDPS in the PAH rat model; hence, we inferred that the inhibition of FDPS might affect pulmonary artery endothelial function. In our study, we found that FDPS inhibitor ibandronate reduced the thickness of the pulmonary artery muscle layer; however, they did not reduce pulmonary artery pressure, nor did they reduce right heart hypertrophy. Arguably, reduced pulmonary remodeling should improve hemodynamics. We think that the complex pathologic causes of pulmonary hypertension and the timing of ibandronate use may explain this paradoxical phenomenon. After 2–3 weeks of monocrotaline intervention, pulmonary EC showed swelling and necrosis, and the loss of EC barrier protection function resulted in protein leakage and interstitial edema. Many vasoactive factors and growth factors directly act on pulmonary artery smooth muscle cells (PASMCs) and fibroblasts, promoting their proliferation and collagen deposition (Nogueira-Ferreira et al. 2015). Additionally, EC synthesizes and releases a variety of vasoactive substances, such as vasoconstrictor endothelin-1, vasodilator nitric oxide and prostacyclin. An uncontrolled balance between these vasoactive factors increases vascular tone and promotes pulmonary EC and PASMC dysfunction (Gomez-Arroyo et al. 2012). Ibandronate therapy was performed exactly 2 weeks after MCT injection. Therefore, with a decrease in the thickness of the arteriolar middle layer in the lung tissue sections, the pathological environment induced by MCT was not completely reversed, and the continuous constriction of pulmonary vessels caused by various abnormal factors would also maintain abnormal hemodynamics. A number of studies have confirmed the function of autophagy in facilitating the progression of PAH, but their conclusions are not completely consistent and are still controversial. In human PAH lung tissue samples, autophagy was elevated. (Lee et al. 2011b). In a chronic hypoxia mouse model, autophagy was increased (Teng et al. 2012). Beclin1 deficiency promotes pulmonary vascular hyperplasia and endothelial cell proliferation (Lee et al. 2011a). In contrast, inhibition of autophagy hinders the progression of PAH in the MCT-induced rat PAH model (Long et al. 2013). To elucidate the function of autophagy in the pathogenesis of PAH, more studies are needed. In the present study, we observed an increased ratio of LC3b II to LC3b I and an increased percentage of mRFP-LC3 punctate-positive cells in MCTP-treated PAECs, confirming upregulated PAEC autophagy in PAH.

Our study detected a decrease in NO levels after MCTP treatment of PAECs. MCT-induced PAH damages PAECs, resulting in reduced NO synthesis. The normal function of the endothelium maintains the balance between vasoconstrictors and vasodilators. Studies have shown that PAEC damage during PAH leads to uncoupling of eNOS, decreased expression or activity of eNOS, and thus reduced NO production, impaired endothelium-dependent vasodilation, and ultimately increased pulmonary vascular resistance (Klinger et al. 2013). As for NO signal and autophagy, studies have shown that NO negatively regulates autophagy via the JNK1–Bcl-2–Beclin1 and the IKK–AMPK–TSC2–mTOR pathways (Sarkar et al. 2011). This may also suggest that reduced NO may be partly responsible for increased levels of autophagy in PAECs during PAH. Inhibition of endogenous NO synthesis reduced endothelial cell proliferation (Ziche et al. 1994). Changes in autophagy levels also affect endothelial cell proliferation. For example, rapamycin-induced autophagy stimulated proliferation in HUVECs by affecting AMPK/Akt/mTOR signaling (Liang et al. 2018). Therefore, when PAH occurs, the damage to PAECs reduces the production of NO, leading to an increase in its autophagy level, which promotes its proliferation.

**Fig. 6:**
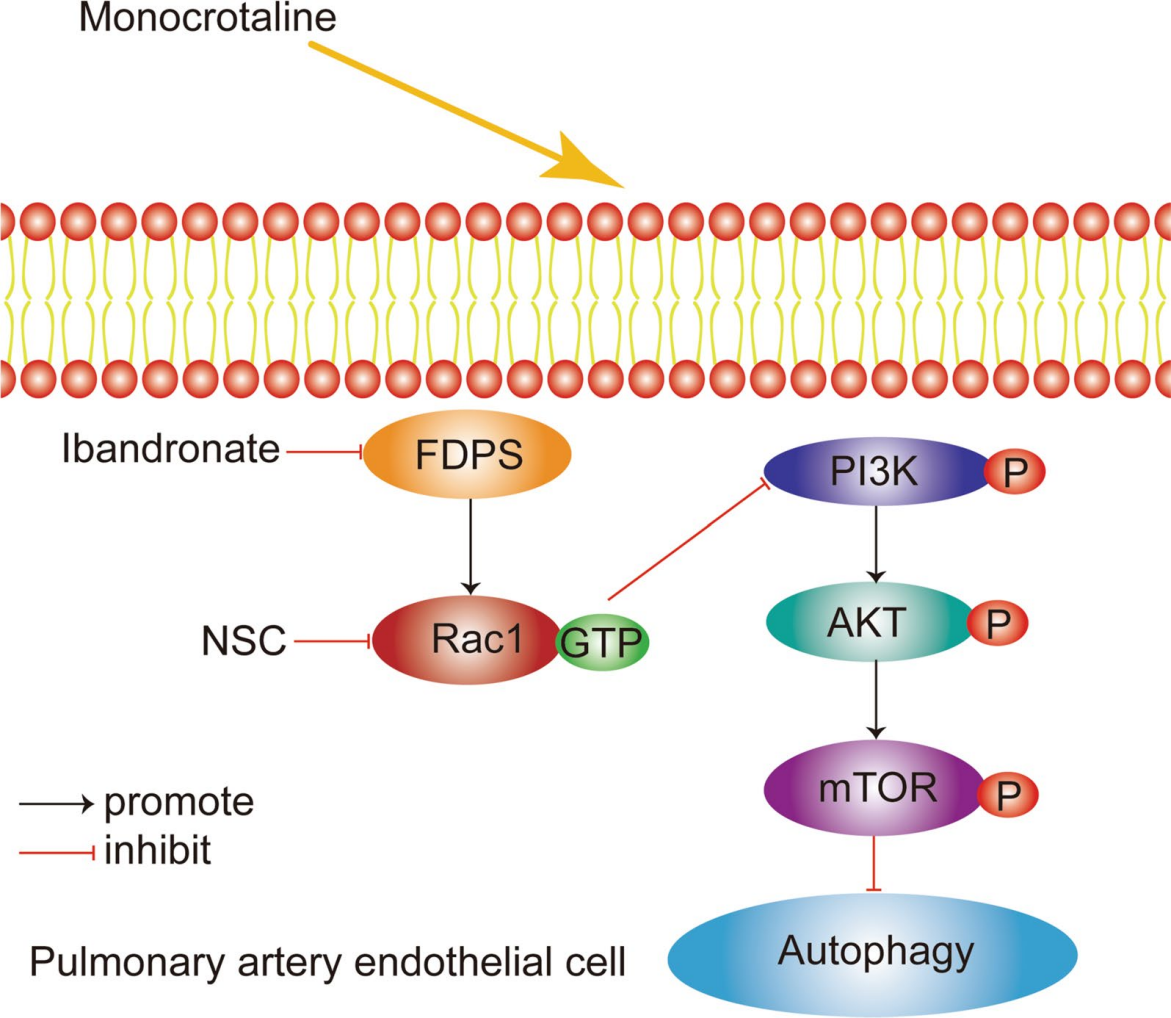
Illustration of the role of FDPS on autophagy in PAECs upon PAH. FDPS expression is up-regulated in pulmonary artery after MCT treatment, which consequently leads to an increase of PAECs autophagy level through the PI3K/AKT/mTOR pathway. Additionally, FDPS may also increase activation of Rac1. Inhibition of FDPS or Rac1 reduces PAECs autophagy and proliferation

MVA pathway intermediate products activated the small G protein. We observed an increase in Rac1 activity in the PAH model. The increase in FDPS, a key enzyme of the mevalonate pathway, explained this finding. Rac1 is a ubiquitously expressed protein, a member of the Rac subfamily of the Rho family of GTPases, which contributes to a diverse array of cellular events, such as the cell cycle, transcription, cell death, cell movement, and oxidoreduction (Burridge and Wennerberg 2004; Bustelo et al. 2007). PI3K activation phosphorylates and activates AKT, and then AKT can activate mTOR. They are involved in various cellular events, such as cell migration, growth, metabolism, longevity, proliferation, immunoreaction and redox reaction. Studies have shown that Rac1 is upstream of AKT and promotes AKT signaling (Kuijk et al. 2008; Lin et al. 2011; Xue et al. 2011). AKT has also been shown to negatively regulate (Kwon et al. 2000; Ozaki et al. 2003) or positively regulate Rac1 activation (Hawkins et al. 1995; Tang et al. 2000; Chung et al. 2001). In the process of activation and modulation of mitochondrial ROS generation, AKT upregulates Rac1 geranylgeranylation and then promotes Rac1 activation (Larson-Casey et al. 2014). In our research, the PI3K/AKT/mTOR pathway was activated in pulmonary artery hypertension, and we believe that Rac1 plays an important role in this process (Fig. 6).

However, due to equipment limitations, we used a homemade PE catheter measuring right ventricular pressure, which is not as sensitive and accurate as Millar Pressure–Volume (PV) catheters. This may result in right ventricular pressure in our rat model of pulmonary hypertension not being as high as has been reported in some literature (Novelli et al. 2020). Due to limited experimental conditions, we selected the usage and dosage of ibandronate in this paper for experiments according to the literature, and the results showed that ibandronate did not significantly improve the hemodynamics and survival rate of mice. Therefore, in further studies, we may be able to explore the preventive and therapeutic effects of ibandronate on PAH by changing the usage and dosage of ibandronate.

## Conclusion

FDPS is important for the proper function of Rac1, which regulates the level of autophagy and proliferation. Therefore, inhibition of FDPS ameliorates endothelial dysfunction, such as inhibition of abnormal proliferation, in PAH, in part due to a reduction in GTP-bound active Rac1 and accompanying reduced autophagy via the PI3K/AKT/mTOR signaling pathway. Therefore, inactivation of FDPS may serve as a potential therapeutic strategy for PAH.

## Supplementary Information

**Additional file 1: Figure S1.** The phenotypes of pulmonary artery hypertension in vivo and in vitro and the changes of other mevalonate pathway enzymes. **Figure S2.** Knockdown of Rac1 gene expression in PAECs by transfected with siRac1.

## Data Availability

The date used to support the findings of this study are available from the first author upon request.

## Abbreviations

PAECs: Pulmonary artery endothelial cells
FDPS: Farnesyl diphosphate synthase
Rac1: Ras-related C3 botulinum toxin substrate 1
PAH: Pulmonary artery hypertension
MCT: Monocrotaline
MCTP: Monocrotaline pyrrole
FPP: Farnesylpyrophosphate
GGPP: Geranylgeranylpyrophosphate
ATG: Autophagy related proteins
IB: Ibandronate
RVSP: Right ventricular systolic pressure
RVH: Right ventricular hypertrophy
LV: Left ventricle
IVS: Interventricular septum
CCK8: Cell Counting Kit‐8
qRT-PCR: Quantitative real-time polymerase chain reaction
MEV: Mevalonate
PI3K: Phosphatidylinositide 3 kinases
mTOR: Mammalian target of rapamycin
