# Supplementary material for: Farnesyl diphosphate synthase regulated endothelial proliferation and autophagy during rat pulmonary arterial hypertension induced by monocrotaline
Source: Mol Med. 2022 Aug 12;28:94. doi: 10.1186/s10020-022-00511-7 (PMC9373289; doi:10.1186/s10020-022-00511-7)
Supplement: Supplementary file 1 — Additional file 1: Figure S1. The phenotypes of pulmonary artery hypertension in vivo and in vitro and the changes of other mevalonate pathway enzymes. Figure S2. Knockdown of Rac1 gene expression in PAECs by transfected with siRac1. [file 10020_2022_511_MOESM1_ESM.docx]

**
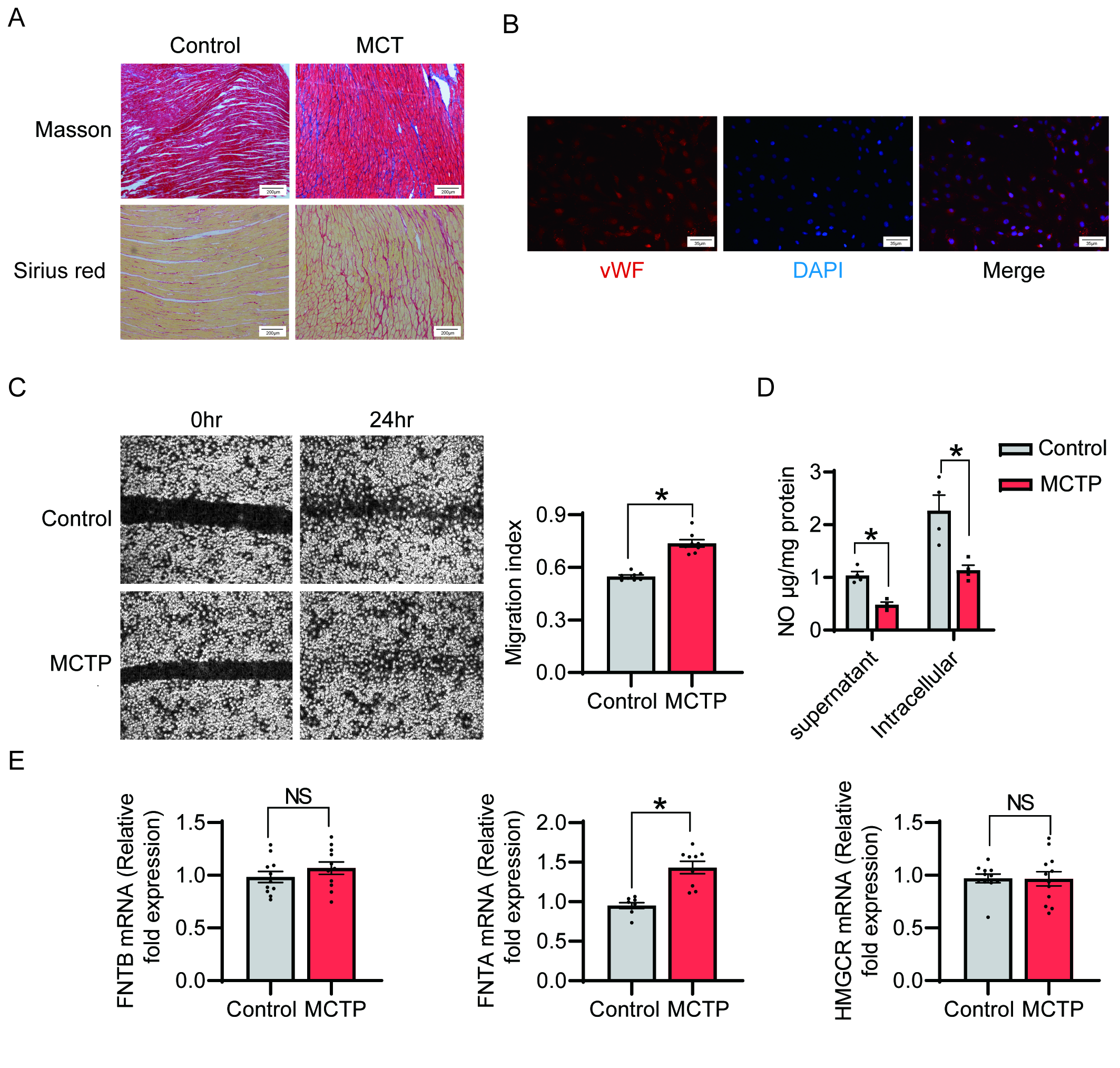
 Figure S1:The phenotypes of pulmonary artery hypertension in vivo and in vitro and the changes of other mevalonate pathway enzymes.**

1. Masson and Sirius red staining of myocardium from MCT induced PAH rat. Significant fibrosis is seen. The representative images are shown. Scale bar = 200 μm. **(B)** Fluorescence immunocytochemical identification of von Willebrand factor was shown in red in PAECs. The nuclear marker Brdu was shown in blue. **(C)** MCTP increased the migration of PAECs. The scratch assay following 50uM MCTP treating for 24 hours in PAECs. Cells covered the plate and wounded by a sterile 200 μl pipetting tip. The ability of cell migration was monitored with an inverted microscope equipped with a digital camera. Representative photomicrographs were shown in left. The index of migration was calculated and showed in right. n = 5 per group. **(D)** MCTP reduced the NO production in PAECs. NO assay was performed to detect NO concentrations in culture medium and cell lysates. Relative NO concentration in cell lysates was normalized based on total cellular protein concentrations. n = 4 per group. **(E)** The mRNA level of FNTA, FNTB and HMGCR in PAECs after 50 μm MCTP treatment for 24 hours was detected. n = 8-12 per group. Data are represented as mean ± SEM. All experiments were independently replicated in triplicate. * p < 0.05. HMGR, 3-hydroxy-3-methylglutaryl-coenzyme; FNTA, farnesyltransferase α; FNTB, farnesyltransferase β.

**
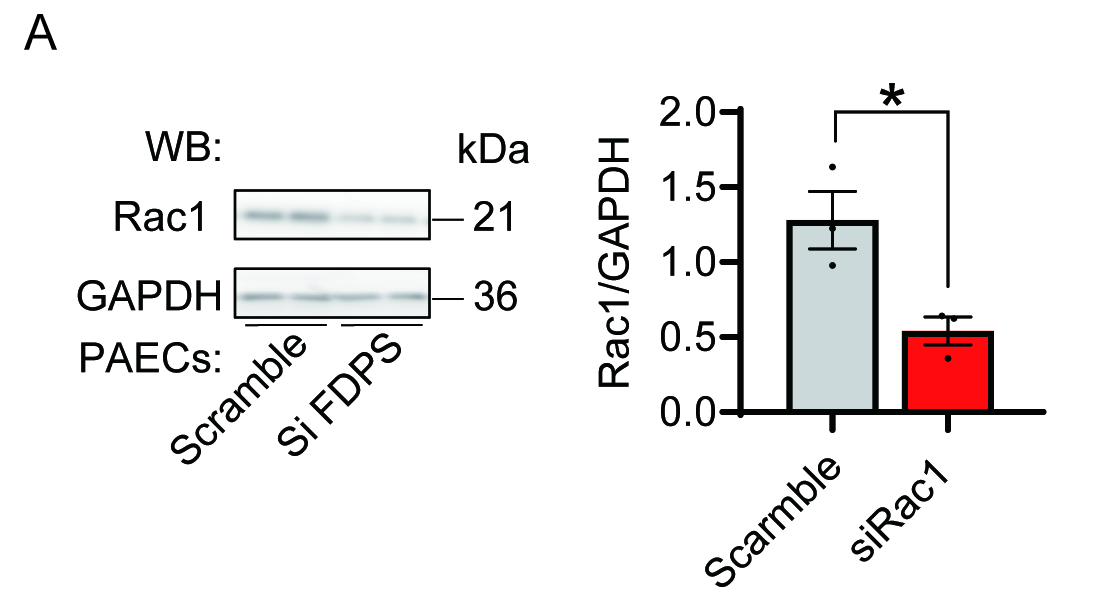
**

**Figure S2: Knockdown of Rac1 gene expression in PAECs by transfected with siRac1.**

**(A)** Immunoblot analysis for FDPS. GAPDH was used as a loading control. n = 3 per group. Data are represented as mean ± SEM. All experiments were independently replicated in triplicate. * p < 0.05.
